# Editorial: The 24-hour activity cycle and cognitive health: how are physical activity, sedentary behavior, and sleep interactively associated with cognitive health across the lifespan?

**DOI:** 10.3389/fnhum.2023.1248262

**Published:** 2023-07-26

**Authors:** Ryan Stanley Falck, Teresa Liu-Ambrose, Jannique Van Uffelen, Helen Macpherson, David X. Marquez, Paul Gardiner, Hans H. C. M. Savelberg

**Affiliations:** ^1^School of Biomedical Engineering, The University of British Columbia, Vancouver, BC, Canada; ^2^Aging, Mobility, and Cognitive Neuroscience Laboratory, Department of Physical Therapy, The University of British Columbia, Vancouver, BC, Canada; ^3^Djavad Mowafaghian Centre for Brain Health, Vancouver Coastal Health Research Institute, Vancouver, BC, Canada; ^4^Centre for Aging Solutions for Mobility, Activity, Rehabilitation and Technology (SMART) at Vancouver Coastal Health, Vancouver Coastal Health Research Institute, Vancouver, BC, Canada; ^5^Department of Movement Sciences, KU Leuven, Leuven, Belgium; ^6^School of Exercise and Nutrition Sciences, Faculty of Health, Deakin University, Burwood, NSW, Australia; ^7^Department of Kinesiology and Nutrition, School of Applied Health Sciences, University of Illinois Chicago, Chicago, IL, United States; ^8^School of Public Health, Faculty of Medicine, University of Queensland, Brisbane, QLD, Australia; ^9^Department of Human Biology and Movement Sciences, School of Nutrition and Translational Research in Metabolism, Faculty of Health, Maastricht University, Maastricht, Netherlands

**Keywords:** physical activity, sedentary behavior, sleep, cognitive health, lifespan

## Introduction

Worldwide, one new case of dementia is detected every 3 s (Alzheimer's Disease International, 2015), with no effective drug therapy available presently. However, up to 40% of global dementia cases can be prevented by modifiable lifestyle factors (Livingston et al., 2020). Identifying effective modifiable lifestyle-strategies are thus not only greatly needed for dementia prevention, but for cognitive health across the lifespan (Landry and Liu-Ambrose, 2014).

Physical activity, sedentary behavior, and sleep are three behaviors in which all humans engage in daily and may play a critical role in maintaining cognitive health (Syväoja et al., 2013; Mellow et al., 2022). Collectively, these behaviors are referred to as the 24-h activity cycle (24HAC). However, few studies have examined the inter-relationships of these behaviors with cognitive health.

This Research Topic explores how the 24HAC interacts with cognitive health across the lifespan. We now highlight the main findings of each contribution to this article collection.

## Main findings

In a longitudinal analysis from the United States of data from the National Institute of Child Health and Human Development (NICHD) Longitudinal Study of Early Child Care and Youth Development (*N* = 1,364), Ramer et al. examined whether childhood 24HAC behaviors were associated with executive function or academic performance in adolescence. Self-reported sleep quality, recreational screen time, and actigraphy-assessed physical activity were collected during grade 5 when the children were ~11 years; executive function and academic performance were collected during grade 9 at age ~15 years. Using structural equation modeling, the authors found: (1) adolescent executive functions were negatively affected by greater childhood recreational screen time and positively affected by better sleep quality; and (2) the negative association between childhood recreational screen time and adolescent executive functions were attenuated by greater childhood physical activity. Interestingly, childhood physical activity was negatively associated with adolescent executive functions; however, this relationship was mitigated by greater sleep.

In a cross-sectional study with 76 Japanese community-dwelling adults aged > 60 years, Hyodo et al. examined the associations between 24HAC composition and executive functions. Physical activity and sedentary behavior were assessed by hip-worn actigraphy, while sleep duration was indexed using self-report. Using compositional data analysis, the authors found longer time spent in light intensity physical activity was associated with better executive performance. In addition, reallocating 30 min/day of sedentary behavior or sleep to light physical activity was associated with better executive performance.

Wu et al. applied three analytic methods (i.e., isotemporal substitution, compositional data analysis, and latent profile analysis) to data from the Adult Changes in Thought study (United States; *N* = 1,034; aged 65+ years) and examined the interactive relationships of 24HAC behaviors with global cognitive function. Physical activity and sedentary behavior were assessed by actigraphy, while sleep was estimated as self-reported time in bed. No significant associations were found across all three analytic methods in this cross-sectional study.

Mellow et al. cross-sectionally examined the associations between 24HAC composition and cognitive function among 384 Australian healthy adults, aged 60–70 years. The 24HAC was assessed by actigraphy and by questionnaires. Cognitive measures included global cognition, memory, executive functions, and processing speed. Using compositional data analysis, no association between 24HAC composition and cognitive function was found.

Hicks et al. cross-sectionally explored how chronotype—an individuals' preferred activity and sleep pattern—and physical activity were associated with cognitive performance (i.e., verbal memory, attention, and executive function) in 153 American adults aged > 60 years, with and without self-reported sleep disorders. Wrist-worn actigraphy assessed physical activity and chronotype. Using multivariate analysis of covariance, no differences in physical activity or cognitive performance between different chronotypes were found.

## Summary

The results indicate that there is still much we do not understand about the 24HAC and cognitive health relationship. It is difficult to broadly characterize the 24HAC and cognitive health relationship at this time given that: (1) most of the evidence is cross-sectional; and (2) there are few studies on the 24HAC and cognitive health relationship in children, adolescents, younger, or middle-aged adults.

Nevertheless, there are several conclusions we can draw from these studies. Notably, time-use composition of the 24HAC does not appear to be strongly associated with cognitive function in healthy older adults. Wu et al., Mellow et al., and Hicks et al. all found no association between 24HAC composition and cognitive function in healthy older adults; only Hyodo et al. found an association between 24HAC composition and cognitive function. It is possible that time-use composition fails to capture aspects of the 24HAC (e.g., sleep quality and architecture) that may be more important for cognitive health.

There is an obvious lack of longitudinal studies in this emerging field of research. Only Ramer et al. used a longitudinal design. We strongly urge that future studies use longer follow-up periods and include wider age-ranges in study samples.

Our article collection mainly includes studies in older adults, but does not include any research among clinical populations at risk for, or living with, cognitive decline or dementia (e.g., mild cognitive impairment or stroke). People living with clinical conditions which are associated with cognitive decline are often less active, more sedentary, and have poorer sleep (Falck et al., 2017, 2019).

Finally, it is unclear what modifiable and non-modifiable moderators and mediators can impact the 24HAC and cognitive health relationship. None of the studies in this Research Topic explored sex differences. Females have twice the risk of dementia (Ferretti et al., 2018) and lifestyle may be a key moderator of dementia risk among females (Barha et al., 2019)—although many have argued that sex-differences in dementia may largely be explainable by the longer life expectancy of females compared with males, and traditionally lower levels of education among women who grew up in times or societies which did not encourage continued education for women (Nicoletti et al., 2023). Sex-differences in Alzheimer's disease pathology accumulation are even less understood, as the data are still limited. Future work is thus still needed to explore whether there are sex-differences in the 24HAC and cognitive health relationship.

## Conclusion

Preserving cognitive abilities across the lifespan is critical for qualify of life and wellbeing. The 24HAC holds promise as a means of maintaining cognitive health from childhood into older adulthood. Future work is needed to identify the temporal 24HAC and cognitive health relationship, as well as understand how different populations, mediators, and moderators such as sex, may impact this relationship across the lifespan.

## Author contributions

RF wrote the first draft of the manuscript. TL-A, JV, HM, DM, PG, and HS each provided critical edits and wrote portions of the manuscript. All authors contributed to the article and approved the submitted version.
